# Cementless bipolar hemiarthroplasty compared with proximal femoral nail anti-rotation of unstable intertrochanteric fractures in senile patients with osteoporosis: a retrospective study

**DOI:** 10.1186/s12891-022-05426-2

**Published:** 2022-05-16

**Authors:** Chengkui Cai, Liying Tian, Zhihui Chen, Pengcheng Sun, Guozhu Wang

**Affiliations:** 1 Department of Orthopedics, The Second Affiliated Hospital of Shaanxi University of Chinese Medicine, Xianyang, 712000 Shaanxi China; 2 Department of Anesthesiology, The Second Affiliated Hospital of Shaanxi University of Chinese Medicine, Xianyang, 712000 Shaanxi China

**Keywords:** Hemiarthroplasty, Proximal femoral nail anti-rotation, Unstable intertrochanteric fracture, Osteoporosis, Elderly

## Abstract

**Background:**

Implant design for the correction of osteoporotic unstable intertrochanteric fractures in elderly patients is a controversial issue. Our study aims to compare the efficacy of PFNA and cementless bipolar hemiarthroplasty (CBH) in treating osteoporotic unstable intertrochanteric fractures in the elderly.

**Methods:**

We retrospectively assessed 70 elderly patients, aged > 70 years old, with intertrochanteric fractures (AO/OTA 31-A2 fractures) from 2014 to 2019. Among them, 34 patients received PFNA and 36 patients received CBH, accompanied with 2-year follow-ups. Additionally, the efficacy difference between the two implants was compared.

**Results:**

Both groups had similar general variables like age, gender, fracture site, degree of osteoporosis, fracture classification, ASA score, basic diseases, preoperative preparation time, anesthesia mode, amount of postoperative blood loss, hospital length of stay, along with postoperative blood transfusions and postoperative complications (*P* > 0.05). Conversely, significant differences were observed among intraoperative variables (amount of blood loss, amount of blood transfusions, operative time, number of intraoperative fluoroscopy), postoperative variables (weight-bearing time out of bed), and Harris hip function score within 12 months of operation (*P* < 0.05).

**Conclusions:**

CBH showed no obvious advantage over PFNA in the perioperative period in elderly patients with osteoporotic unstable intertrochanteric fractures. However, the joint replacement allowed for earlier ambulation after the operation and rapid recovery of the hip joint function.

**Supplementary Information:**

The online version contains supplementary material available at 10.1186/s12891-022-05426-2.

## Background

The therapeutic principle for treating elderly patients with intertrochanteric fractures aims to facilitate the rapid ambulation of patients after surgery, thus reducing the incidence of bed-related complications, while improving the quality of life after injury and prolonging survival time of patients. Intramedullary fixation is generally recommended for the correction of intertrochanteric fractures [1]. However, in elderly patients with osteoporotic intertrochanteric fractures, due to basic diseases and severe osteoporosis, early ambulation after intramedullary fixation often increases the risk of complications (e.g., prosthesis loosening, peri-prosthetic fracture, and bone nonunion), which results in the failure of the entire treatment plan [2–4]. Hence, the treatment strategy in elderly patients is to apply joint replacement, safely and effectively, thereby achieving early ambulation after the operation [5]. However, there are no clear conclusions on the superiority of joint replacement. This study retrospectively assessed 70 elderly patients with osteoporotic intertrochanteric fractures, who underwent proximal femoral nail anti-rotation (PFNA) or cementless bipolar hemiarthroplasty (CBH) from 2014 to 2019. The collected data were subsequently systematically analyzed and compared, providing a reference for the primary treatment option of elderly patients with osteoporotic intertrochanteric fractures.

## Methods

### Patient information

**Fig. 1:**
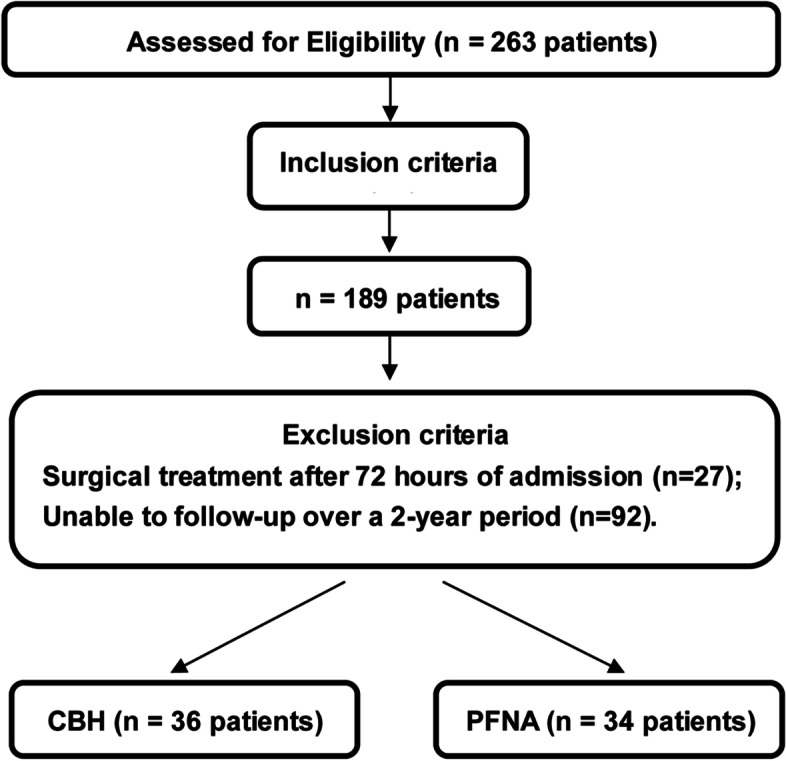
Case screening flow chart

From June 2014 to June 2019, 263 patients with an unstable intertrochanteric femoral fractures treated by the same surgical team were retrieved from the database of our hospital, and our work was approved by our institutional review board. The study inclusion criteria were as follows: The average age of the patients was more than 70 years old; they were able to walk independently before the fracture, and all fractures were caused by low-energy injuries; all patients underwent bone mineral density examination (dual-energy X-ray absorptiometry) upon admission; they received preoperative anteroposterior pelvic and lateral film of the affected hip joint, CT examination, and three-dimensional reconstruction of the bilateral hip joint; intertrochanteric fracture affecting one side, without additional fractures; no absolute contraindication for the operation; no mental disorders; as well as normal erythrocyte sedimentation rate and high-sensitivity C-reactive protein. After screening for inclusion criteria, 74 patients were screened and 189 patients remained. The following patients were excluded from the study: surgical treatment after 72 hours of admission; unable to follow-up over a 2-year period. After screening for exclusion criteria, 70 cases were eligible for this study (Fig. 1). The surgical methods followed the wishes of the patients and their families. Among all patients, 36 cases underwent CBH (the CBH group) and 34 cases underwent PFNA (the PFNA group), with a 2-year follow-up period. The preoperative variables between the two groups are presented in Table 1. There was no difference in the prophylactic antibiotic usage between the two groups. The affected limbs were placed on the Braun’s splint for ankle traction before the operation. All patients in both groups were treated with barotherapy and Rivaroxaban to prevent perioperative thrombosis. Rivaroxaban was administered orally 10 mg, once a day. The drugs were discontinued from 12 hours before the operation to 6 hours after the operation. The drugs were continued from 6 hours after the operation to 35 days after the operation. The following clinical data were collected prior to the operation: age, gender, fracture site, degree of osteoporosis, fracture classification (AO classification), American Society of Anesthesiologists (ASA) score, preoperative preparation time, and basic diseases. The following intraoperative variables were documented: anesthesia mode, amount of blood loss, amount of blood transfusions, number of blood transfusions, operation time, and number of intraoperative fluoroscopies. Lastly, the following postoperative variables were collected: amount of blood loss, amount of blood transfusions, number of blood transfusions, weight-bearing time out of bed, hospitalized days, complications before discharge, complications after discharge, and Harris hip function score at 1.5, 3, 6, 12, 18, and 24 months after the operation. All operations were performed within 72 hours of admission. The same surgical team performed both types of operations, and the surgeon carried out detailed preoperative planning and preparation for both implants. PFNA prosthesis was purchased from the DePuy Synthes Co., Ltd. and CBH prosthesis was purchased from the Beijing Chunli Zhengda Co., Ltd.

**Table 1:** Baseline characteristics of the two groups included in this study

	CBH	PFNA	Statistics	*P* value
Age (years)^a^	82.19 ± 3.96	80.88 ± 4.90	t = 1.235	0.221
Osteoporosis (T value)^a^	−3.53 ± 0.75	−3.77 ± 0.90	t = 1.229	0.223
Preoperative preparation time (hours)^a^	57.33 ± 11.87	54.35 ± 14.84	t = 0.931	0.355
Gender (male/female), n	16/20	16/18	χ^2^ = 0.048	0.826
Fracture site (left/right), n	17/19	20/14	χ^2^ = 0.944	0.331
AO classification, n				
31-A2.2	17	22	χ^2^ = 2.166	0.141
31-A2.3	19	12		
ASA score, n				
III	15	16	χ^2^ = 0.206	0.650
IV	21	18		
Basic diseases, n				
Respiratory	7	6	χ^2^ = 0.037	0.847
Cardiovascular	14	14	χ^2^ = 0.038	0.845
Urinary	3	2	χ^2^ < 0.001^b^	1.000
Neurologic	6	10	χ^2^ = 1.611	0.204
Digestive	3	5	χ^2^ = 0.213^b^	0.644
Endocrinium	9	8	χ^2^ = 0.021	0.886
Genital	2	3	χ^2^ = 0.004^b^	0.947

^a^Data are presented as mean ± standard deviation. ^b^Using chi-squared test with Yates’ correction. t: Student’s t-test. χ^2^: Chi-squared test. *ASA* American Society of Anesthesiologists

### Implant

#### PFNA

**Fig. 2:**
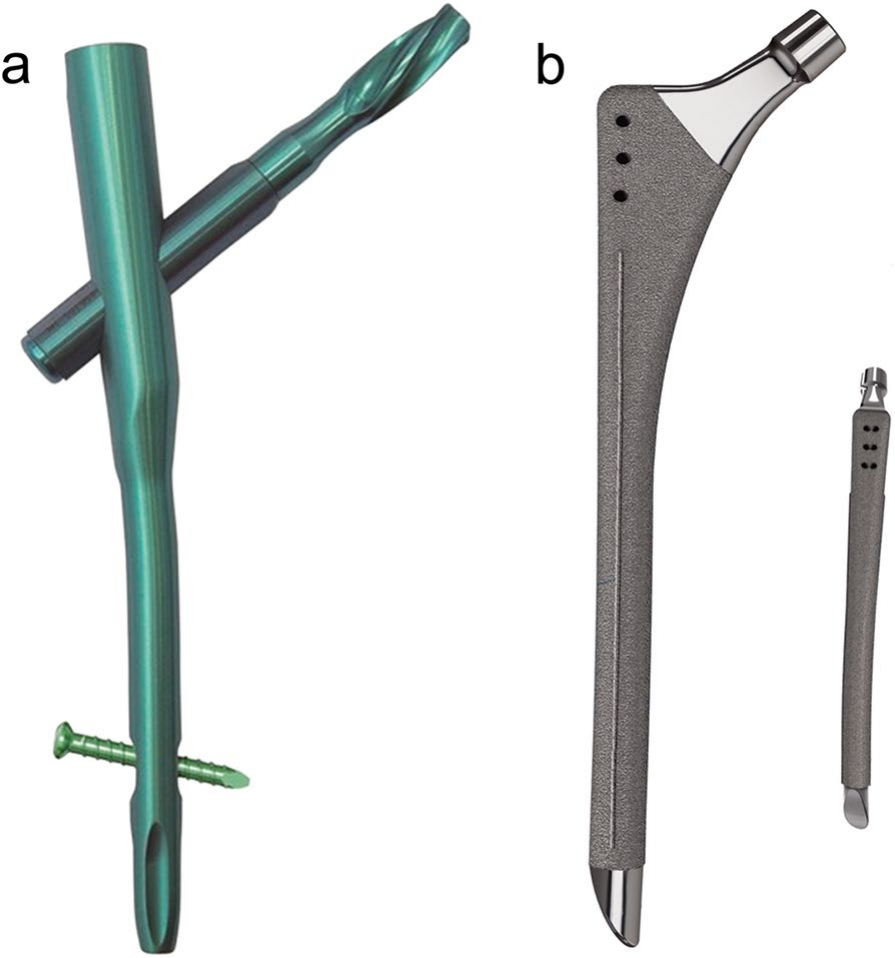
**a** The schematic sketch of PFNA prosthesis; **b** The schematic sketch of CBH prosthesis

The modified Asian PFNA-II-type hollow prosthesis from the DePuy Synthes was used, due to its suitability for the shape of the Chinese femur. The prosthesis was made of titanium alloy. The lateral wall of the main nail was treated as planarization to reduce compression on the lateral wall of the greater trochanter during nail placement. The valgus angle of the main nail was changed to 5^°^, with an anteversion angle of 10^°^. The length was adjusted to 170, 200, and 240 mm and the distal diameter was set to 9–12 mm. The length of hip nail blade was 75–120 mm. In this study, a static locking mode was used in all distal interlocking nails (Fig. 2a).

#### CBH

The 160-type hemiarthroplasty prosthesis developed by the Beijing Chunli Zhengda was used. The femoral stem of the prosthesis was a full-coated anatomical lengthened stem. The prosthesis was made of titanium alloy and the surface was sprayed with rough plasma titanium. Spinous protrusions were added on both sides of the prosthesis to enhance the anti-rotation ability. The distal stem tip of the prosthesis was designed to be eccentric to avoid impact to the lateral femoral wall. The cervical shaft angle of the prosthesis was 130 °, with an anterior arch of 15 °. The length of the stem was 170, 200, and 240 mm. The proximal part of the stem contained three holes, which was convenient for the reconstruction of the greater trochanter fracture. A good match of more than 6 cm between the prosthesis and bone was required to meet the stability criteria of the initial implantation (Fig. 2b).

### The surgical methods

#### PFNA

**Fig. 3:**
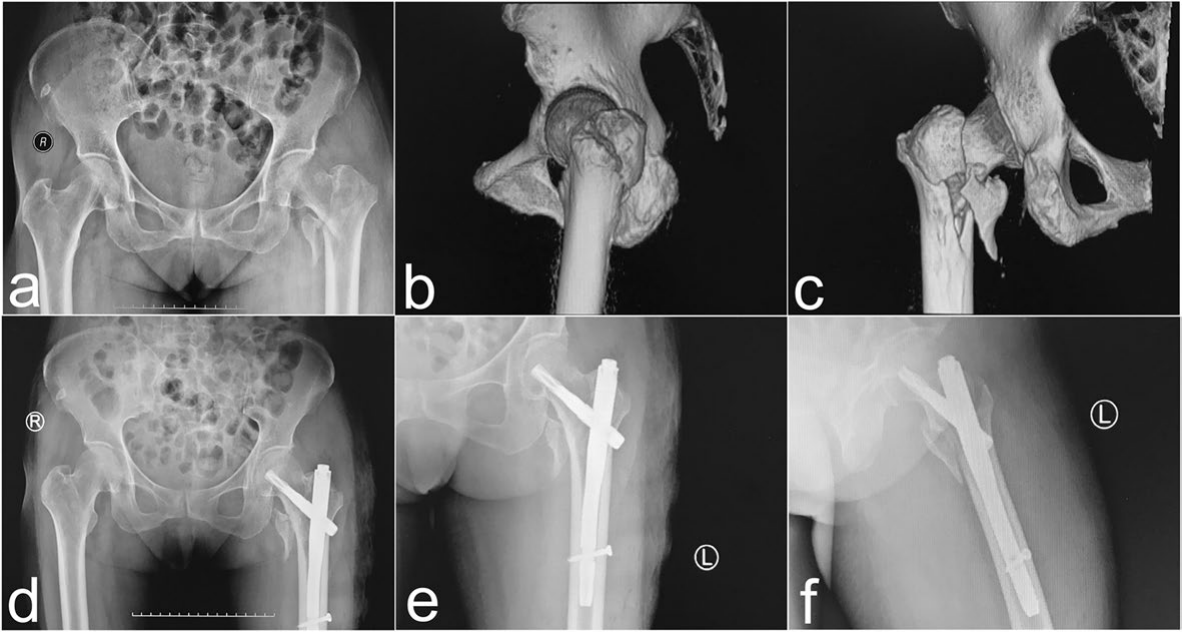
A 76-year-old female patient with left femoral intertrochanteric fracture, caused by a fall while walking (AO classification: 31-A2.2). PFNA was performed. **a** Preoperative pelvic anteroposterior X-ray illustrating a left femoral intertrochanteric fracture. **b** Three-dimensional CT reconstruction of the pelvis showing the lateral image of the left hip joint, and the greater trochanter fracture block is clearly visible. **c** Three-dimensional CT reconstruction of the pelvis demonstrating the anteroposterior image of the left hip joint, and the lesser trochanter fracture block is clearly visible. **d** Postoperative pelvic anteroposterior X-ray images. **e** Postoperative left hip anteroposterior X-ray images. **f** Postoperative left hip lateral X-ray images

PFNA was performed according to the standard procedure provided by DePuy Synthes. Patients with 31-A (2.2–2.3) type fractures were indicated for PFNA. In short, patients were laid supine on the traction bed. After fracture traction closed reduction under C-arm assisted fluoroscopy, the PFNA system was implanted via a minimally invasive incision. The main nail with appropriate length was selected based on the position of the fracture line. Head and tail nails with appropriate length and tail cap with standard length were placed under fluoroscopic guidance. The operation area was flushed with 3 L sterile normal saline. A negative pressure suction tube was placed at the proximal incision of the femoral trochanter and removed 48 hours after the operation (Fig. 3).

#### CBH

**Fig. 4:**
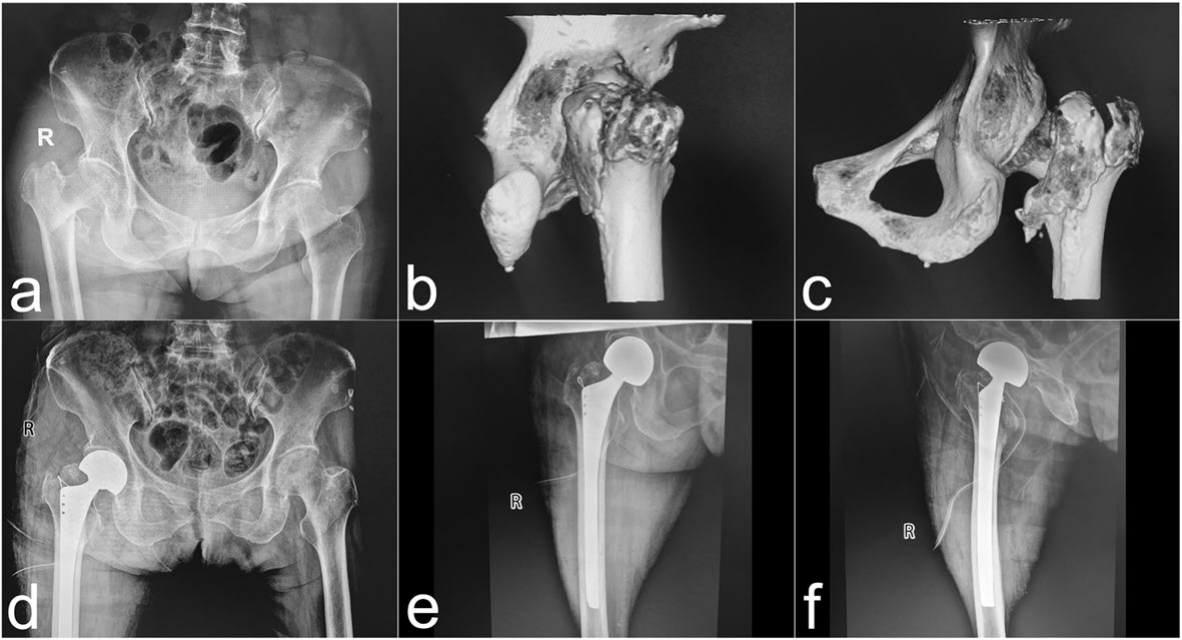
A 77-year-old female patient with right femoral intertrochanteric fracture caused by a fall while walking (AO classification: 31-A2.3). CBH was performed. **a** Preoperative pelvic anteroposterior X-ray illustrating a right femoral intertrochanteric fracture. **b** Three-dimensional CT reconstruction of the pelvis showing the lateral image of the right hip joint, and the greater trochanter fracture block is clearly visible. **c** Three-dimensional CT reconstruction of the pelvis showing the anteroposterior image of the right hip joint, and posterior wall of femoral trochanter and the lesser trochanter fracture blocks are clearly visible. **d** Postoperative pelvic anteroposterior X-ray images. The greater trochanter and the posterior wall of femoral trochanter fracture blocks were bound with steel wire. **e** Postoperative right hip anteroposterior X-ray images. **f** Postoperative right hip lateral X-ray images

CBH was performed via the Moore’s approach. Patients with 31-A (2.2–2.3) type fractures were indicated for CBH. In brief, patients were laid in a lateral decubitus position with the operating area facing up. The femoral neck was cut off and the femoral head was removed. The proximal femur was shaped with a medullary file, and the prosthesis with the appropriate size was placed for model testing. The greater trochanter fracture was reduced and temporarily fixed with Kirschner wire. A vertical line was drawn from the rotation center of the femoral head to the femoral medullary cavity axis to obtain an intersection point. The distance from this point to the trochanter tip along the femoral medullary cavity axis was defined as the relative length of the lower limb. A femoral prosthesis and double-acting head with appropriate size were placed. The greater trochanter fracture was fixed with steel wire and the Kirschner wire was removed. The artificial joint was then reduced and the operation area was flushed with 3 L sterile normal saline, with a tightly sutured articular capsule. A negative pressure suction tube was placed in the incision and removed 48 hours after surgery (Fig. 4).

### Follow-up

The Harris hip function score was conducted via telephonic interview and outpatient follow-up at 1.5, 3, 6, 12, 18, and 24 months post surgery. At each corresponding time point, based on patients’ wishes, patients received anteroposterior pelvic and lateral X-ray of the affected hip joint. An independent radiologist performed the radiologic evaluation. According to the radiologic evaluation, fracture reduction quality was divided into anatomical (< 5° of varus or valgus and/or anteversion or retroversion), acceptable (5° to 10°) or poor (> 10°) groups [6]. Due to the inconsistent willingness of patients in receiving radiological examination at each follow-up time point, the radiologic data was incomplete. Moreover, due to the lack of standard anteroposterior and lateral X-ray images of the hip joints, in most cases, the evaluation of prosthesis stability after the operation could not be carried out. The length of the lower limb, prosthesis loosening, fracture displacement, periprosthetic fracture, fracture nonunion, and joint dislocation were recorded.

### Statistical analysis

The SPSS22.0 software was used for all statistical analyses. Herein, the measured data are statistically expressed in mean ± standard deviation. To compare between groups, an independent t-test was performed for data that met normal distribution and variance homogeneity. If those that did not meet this requirement, the t’ test was performed. The count data was statistically expressed in frequency. For group comparisons, χ [2] test was performed if the total sample size was ≥40 or the number in each cell was ≥5. A continuous corrective χ^2^ test was performed if the total sample size was < 40 or the number in each cell was < 5 and ≥ 1. Fisher exact probability method was performed if the total sample size was < 40 or the number in the cell was 0. *P* < 0.05 was considered significant.

## Results

The preoperative data of the two examined groups are shown in Table 1. Upon statistical analysis, no significant differences were observed in variables between the two groups (*P* > 0.05).

Among the intraoperative data (see Table 2), no significant differences were found in the anesthesia mode (*P* > 0.05), whereas significant differences were observed in other variables (*P* < 0.05). During the operation, the amount of blood loss (236.94 ± 34.13 mL), number of blood transfusions (35), amount of blood transfusions (2.89 ± 0.75 u.) and the operative time (74.89 ± 8.18 min.) in the CBH group were significantly more than the PFNA group. Moreover, the number of intraoperative fluoroscopies (19.29 ± 3.77) in the PFNA group were significantly more than the CBH group.

**Table 2:** Comparison of intraoperative data between the two groups

	CBH	PFNA	Statistics	*P* value
Anesthesia mode, n				
General	12	11	χ^2^ = 0.008	0.930
Regional	24	23		
Blood loss (ml)^a^	236.94 s± 34.13	182.06 ± 42.23	t = 5.993	< 0.001
Transfusion				
No. of units (u)^a^	2.89 ± 0.75	0.71 ± 0.97	t = 10.503	< 0.001
No. of patients, n	35	12	χ^2^ = 27.655^b^	< 0.001
Operative time (minutes)^a^	74.89 ± 8.18	54.06 ± 5.78	t = 12.352	< 0.001
Fluoroscopy^a^	1.08 ± 1.32	19.29 ± 3.77	t = −26.669	< 0.001

^a^Data are presented as mean ± standard deviation. ^b^Using chi-squared test with Yates’ correction. t: Student’s t-test. χ^2^: Chi-squared test

Analyzing the data of patients after the operation and before discharge (see Table 3), significant differences were evident in the postoperative weight-bearing time out of bed (*P* < 0.05). However, no significant difference was observed in the amount of postoperative blood loss, blood transfusions and hospitalization period (*P* > 0.05). The postoperative weight-bearing time out of bed in the CBH group (2.39 ± 0.77 days) was remarkably more than the PFNA group. Comparing the complications after the operation but before discharge, the CBH group experienced less complications than the PFNA group in the number and type of disease. There were 11 events in the CBH group (e.g., atrial fibrillation, ureteral calculi, and urinary retention, deep venous thrombosis), while 23 events occurred in the PFNA group (e.g., pneumonia, coronary heart disease, urinary retention, cerebral infarction, postoperative vomiting, infection and constipation).

**Table 3:** Comparison of data from operation to discharge between the two groups

	CBH	PFNA	Statistics	*P* value
Blood loss (ml)^a^	193.33 ± 142.81	168.82 ± 130.77	t = 0.748	0.457
Transfusion				
No. of units^a^	1.22 ± 1.59	1.12 ± 1.34	t = 0.297	0.768
No. of patients	16	17	χ^2^ = 0.217	0.642
Out of bed time (day)^a^	2.39 ± 0.77	5.82 ± 1.57	t = −11.548	< 0.001
Hospitalized days (day) ^a^	13.22 ± 5.93	10.91 ± 5.61	t = 1.673	0.099
Complications before discharge				
Respiratory	0	3		0.109^b^
Cardiovascular	1	4	χ^2^ = 0.990^c^	0.320
Urinary	2	3	χ^2^ = 0.004^c^	0.947
Neurologic	4	3	χ^2^ < 0.001^c^	1.000
Digestive	0	2		0.232^b^
Thrombus	2	7	χ^2^ = 2.313^c^	0.128
Infection	2	1	χ^2^ < 0.001^c^	1.000

^a^Data are presented as mean ± standard deviation. ^b^Using Fisher probabilities in 2 × 2 table. ^c^Using chi-squared test with Yates’ correction. t: Student’s t-test. χ^2^: chi-squared test

Comparing complications after discharge revealed significantly different types of complications between the two groups. The CBH group experienced 4 cases of unequal lower limb lengths, 2 cases of delayed incision healing, and 2 cases with fracture nonunion. Patients in the PFNA group, on the other hand, experienced 5 cases of prosthesis loosening, 3 cases of refracture, and 3 cases of reoperation. However, after statistical analysis, there was no significant difference in complications between the two groups (*P* > 0.05, see Table 4).

**Table 4:** Complications of the two groups from the time of discharge to two years

	CBH	PFNA	Statistics	*P* value
Delayed incision healing	2	0		0.493^a^
Dearticulation	1	0		1^a^
Limb length inequality	4	0		0.114^a^
Prosthetic loosening	1	5	χ^2^ = 1.835^b^	0.176
Prosthesis fracture	0	1		0.486^a^
Nonunion	2	1	χ^2^ < 0.001^b^	1
Reoperation	0	3		0.109^a^
Refracture	1	3	χ^2^ = 0.329^b^	0.350
Thrombus	2	1	χ^2^ < 0.001^b^	1

^a^Using Fisher probabilities in 2 × 2 table. ^b^Using chi-squared test with Yates’ correction. χ^2^: chi-squared test

Comparison of the Harris hip function scores at 1.5, 3, 6, 12, 18, and 24 months after operation revealed that patients in the CBH group had enhanced hip function at 1.5–12 months after the surgery (*P* < 0.05). Nevertheless, no obvious difference was observed in the joint function score between the two groups from 18 to 24 months (*P* > 0.05, see Table 5).

**Table 5:** Harris hip function scores of the two group

Time (month)	CBH	PFNA	t value	*P* value
1.5	49.98 ± 21.13	31.93 ± 11.01	4.519	< 0.001^a^
3	66.57 ± 16.66	47.35 ± 12.70	5.403	< 0.001^a^
6	75.52 ± 11.96	60.95 ± 14.94	4.515	< 0.001^a^
12	79.71 ± 11.25	73.46 ± 10.70	2.377	0.020^a^
18	81.93 ± 9.87	79.56 ± 10.74	0.965	0.338
24	80.63 ± 10.16	80.85 ± 11.82	−0.085	0.993

Data are presented as means ± standard deviation. t: Student’s t-test. ^a^Statistically significant between the two groups (*P* < 0.05)

## Discussion

Due to severe osteoporosis and poor muscle elasticity, fractures in the elderly are generally comminuted. In addition, the elderly tends to have numerous basic diseases, and additional physical weakness. Hence, if an appropriate treatment is not designed, chances of remedying the fracture are extremely low. Therefore, elderly patients with intertrochanteric fractures are usually referred to as end-of-life fractures [7]. The core purpose of this kind of fracture treatment is to provide stable and effective bony support for the limbs, which can help the patients recover their walking ability as soon as possible. In these cases, fracture healing is not emphasized. Currently, intramedullary fixation is the preferred treatment for this kind of fracture [8–10]. Within the intramedullary fixation system, PFNA, due to its minimal invasiveness, exhibits excellent biomechanical and stable fixation outcomes, which are highly preferred in case of osteoporotic unstable intertrochanteric fractures [11]. However, in case of the 31-A (2.2–2.3) type elderly patients with intertrochanteric fractures, bone fragments at the greater and lesser trochanters cause destruction and loss of important mechanical bone structures, thus affecting anti-pressure, tension, rotation and inversion at the femoral trochanter. Maintenance of the main fracture blocks via internal fixation alone cannot meet the corresponding mechanical requirements. Furthermore, it is difficult to reduce the fracture during the operation and, in case of severe osteoporosis, the chances of nail loosening and cutting out is markedly elevated [12–14]. Prior reports suggested that the failure rate of a femoral proximal intramedullary nail in treating intertrochanteric fractures is between 7.1–12.5% [15, 16]. Patients with unstable intertrochanteric fractures treated with PFNA need to walk without weight bearing in the early postoperative period. The upper limb strength of the elderly is weak and it is difficult to walk even with the help of double crutches or walking aids. Moreover, the potential fear of internal fixation loosening leads to the tendency of long-term bed rest, which increases the probability of bed-related complications. Thus, PFNA cannot achieve the core purpose of this kind of fracture treatment [3, 17]. In contrast, CBH can quickly provide appropriate stability for the mechanical structures around the hip joint. Therefore, patients are able to become mobile early after operation and walk with weight using the affected limb, which, in turn, significantly improves the postoperative experience of patients and achieves the purpose of helping patients gain mobility quickly after operation [18, 19]. CBH is also highly recommended by many doctors [20–22]. Kim et al. conducted a prospective clinical trial on elderly patients with unstable intertrochanteric fractures and compared the therapeutic effect of the long-stem cementless artificial bipolar femoral head prosthesis and PFNA. They found that joint replacement could help the patients regain mobility earlier [23]. Similarly, Broos et al. reported a follow-up of 94 elderly patients treated with artificial bipolar femoral head replacement and found that the average operative time of the bipolar femoral head replacement group was shorter, the mortality was lower, and the prognosis was better [5]. Likewise, Haentjens et al. reported that patients with comminuted femoral intertrochanteric fractures and severe osteoporosis can benefit from hemiarthroplasty. Hence, CBH was recommended for elderly patients with severe osteoporosis, poor prognosis after the internal fixation, short-life expectancy and poor stability of comminuted fractures [19].

Our study retrospectively investigated the difference between CBH and PFNA in treating elderly patients with osteoporotic unstable intertrochanteric fractures. Our analysis revealed that the intraoperative blood loss, intraoperative blood transfusions in the CBH group was considerably more than the PFNA group. However, there was no difference in the amount of postoperative blood loss and transfusions between the two groups, indicating that the amount of blood loss and the need for blood transfusions in the CBH group was more than that of the PFNA group during the perioperative period. However, patients in the PFNA group required multiple intraoperative fluoroscopies. The number of intraoperative fluoroscopies in the PFNA group were more than that of the CBH group, and the operative time was longer than that of the CBH group. Patients in the CBH group were able to become mobile significantly faster than in the PFNA group. The types of postoperative bed-related complications were visibly different between the two groups. CBH group, for instance had 11 events, whereas the PFNA group had 23. Comparing between the long-term complications after discharge, the main complications in the CBH group were the unequal length of lower limbs, fracture nonunion, and delayed incision healing, while the main complications in the PFNA group were prosthesis loosening, refracture, and reoperation. The postoperative hip joint Harris score revealed that the CBH group score was better than the PFNA group within 12 months of operation, indicating that the CBH surgery achieves earlier joint motion function. However, there was no significant difference in the score between the two groups after 18 months, indicating that CBH and PFNA achieve similar long-term effects on joint motion function 18 months after operation. Based on the above results, the patients in the PFNA group experienced less blood loss and less blood transfusions during the perioperative period. Alternately, the patients in the CBH group experienced reduced operative time and less intraoperative fluoroscopy. Moreover, patients in the CBH group achieved early mobilization, and exhibited enhanced hip joint motion within 12 months after operation.

PFNA is a minimally invasive incision that causes less bleeding during surgery. However, based on the characteristics of repeated fluoroscopy in minimally invasive surgery, it can prolong the operative time, particularly when radiation is refused. Unstable intertrochanteric fractures with severe osteoporosis can significantly increase chances of internal fixation loosening, which is the main reason why most patients opt against walking, even after receiving medical suggestion to walk with two crutches. For unstable intertrochanteric fractures, the basic principle of the postoperative functional exercise is to conduct early out-of-bed activity as soon as possible, but the affected limb cannot bear the weight entirely. As such, the patient carries weight on one leg and walks with crutches or walking aids. Patients with weak upper limb strength or poor body balance ability, are unable to implement this exercise plan. Hence, many patients remain in bed for a long time after PFNA operation [24]. Unfortunately, this increases the probability of bed-related complications, medical costs, and prolong hospitalization days. CBH therapy can provide a stable load-bearing joint in the early postoperative period and the patients can therefore boldly walk with both lower limbs, which greatly reduces the pressure of postoperative exercise. Most of the elderly are able to get out of bed and walk autonomously with the aid of instruments. It is, however, challenging for the affected limb to gain early weight-bearing ability after CBH therapy. Firstly, enough initial stability needs to form between the prosthesis and the bone. Secondly, the reduction and fixation of greater and lesser trochanter fractures needs to be carried out. Finally, the length of lower limbs needs to be restored [25, 26]. To achieve the above three purposes, joint surgeons need to study and practice for a long time. It is difficult to obtain enough stable interface between the prosthesis and bone with conventional femoral stem prosthesis. Therefore, the lengthened anatomical handle of the medullary cavity is selected, which can achieve early stable connection by pressing the distal end coat of the stem with the distal end of the fracture and the isthmus of the medullary cavity. It has the advantage of avoiding bone contact at the fracture site whilst avoiding bone cement-induced complications [27]. However, with this procedure, many cancellous bones in the proximal femurare destroyed and intraosseous blood supply in the proximal femur are hindered to a certain extent. There also exists a certain risk of stress-induced bone resorption and fracture nonunion at a later stage. In addition, the possibility of repeat operation can greatly increase with the failure of the first operation. Meanwhile, it is crucial for the early postoperative joint movement to reset the greater and lesser trochanter fractures after the prosthetic test [28]. Studies have revealed that greater than 2 cm displacement of the greater trochanter fracture fragments can lead to an apparent abductor weakness [29]. Sound reduction and fixation of greater and lesser trochanter fractures can further induceenhanced muscle strength of the hip flexion, abduction, and external rotation, and requires joint surgeons with excellent good fracture anatomical reduction and fixation skills. Furthermore, the fracture blocks must be reduced and fixed, without excessive dissection of the muscle attachment points, and a steel wire or binding band must be employed for winding and fixation. In this study, only the greater trochanter fractures were reduced and fixed in both groups, while the lesser trochanter fractures were left untreated. The surgical incision for the lesser trochanter fracture usually cleaves off part of the external rotation muscles of the hip joint. This, in turn, can weakens the hip external rotation muscle strength post surgery. Moreover, excessive stripping of the posterior incision can raise the risk of postoperative joint dislocation. The iliopsoas muscle attached to the lesser trochanter is one of the most powerful hip flexors. The binding of the steel wire in this region usually fails to resist muscle traction, and, therefore, leads to the failure of reduction and fixation. Additionally, excessive wire binding can also affect the blood supply of the proximal femur. Interestingly, hip flexion can also be compensated by other muscles. In our study, the lesser trochanter was not reset and fixed, based on the above advantages and disadvantages. From the perspective of a comparative study, both CBH and PFNA therapies performed efficacious non-interference treatment of the lesser trochanter. Differences in other aspects can be more specifically compared under the same conditions. Finally, it is more difficult to control the lower limb length during the operation for patients with both greater and lesser trochanter fractures. Hence, the feasible procedure would be to reset and fix the greater trochanter in advance, after the placement of the femoral stem. Generally, the relative position between the rotation center of the prosthesis and the greater trochanter apex of the femur is used for the evaluation of the lower limb length [30]. An equal length of lower limbs is the premise for mobility in patients after surgery. Post operation, patients with significantly different lengths of lower limbs often encounter an inferior walking experience.

There were certain limitations in our retrospective study. Firstly, the number of cases in this study was insufficient and unequal in both groups. Also, the difference in postoperative complications was not statistically significant in our analysis, which is inconsistent with the research conclusions of other scholars. Secondly, the follow-up time was limited, only 2 years. Hence, a long-term evaluation of postoperative complications like osteonecrosis of the femoral head, joint prosthesis wear, and traumatic arthritis, were not statistically analyzed.

## Conclusion

Compared to the PFNA therapy, patients treated with CBH experienced shorter operative time, fewer fluoroscopy evaluations, and more blood loss and transfusions. No significant difference was observed in the hip joint motion function between the two methods after 18 months. However, patients in the CBH group were able to be mobile earlier after surgery and exhibited better joint motion function within 12 months of operation. Based on these results, we propose that CBH can provide faster recovery in elderly patients with osteoporotic unstable intertrochanteric fractures.

## Supplementary Information

**Additional file 1.**

**Additional file 2.**

## Data Availability

The data and materials are available from the medical records department of the Second Affiliated Hospital of Shaanxi University of Chinese Medicine. The datasets used and analysed during the current study are available from the corresponding author on reasonable request.

## Abbreviations

CBH: Cementless bipolar hemiarthroplasty
PFNA: Proximal femoral nail anti-rotation
ASA: American Society of Anesthesiologists
