# Supplementary material for: Cementless bipolar hemiarthroplasty compared with proximal femoral nail anti-rotation of unstable intertrochanteric fractures in senile patients with osteoporosis: a retrospective study
Source: BMC Musculoskelet Disord. 2022 May 16;23:461. doi: 10.1186/s12891-022-05426-2 (PMC9112522; doi:10.1186/s12891-022-05426-2)
Supplement: Supplementary file 1 — Additional file 1. [file 12891_2022_5426_MOESM1_ESM.docx]

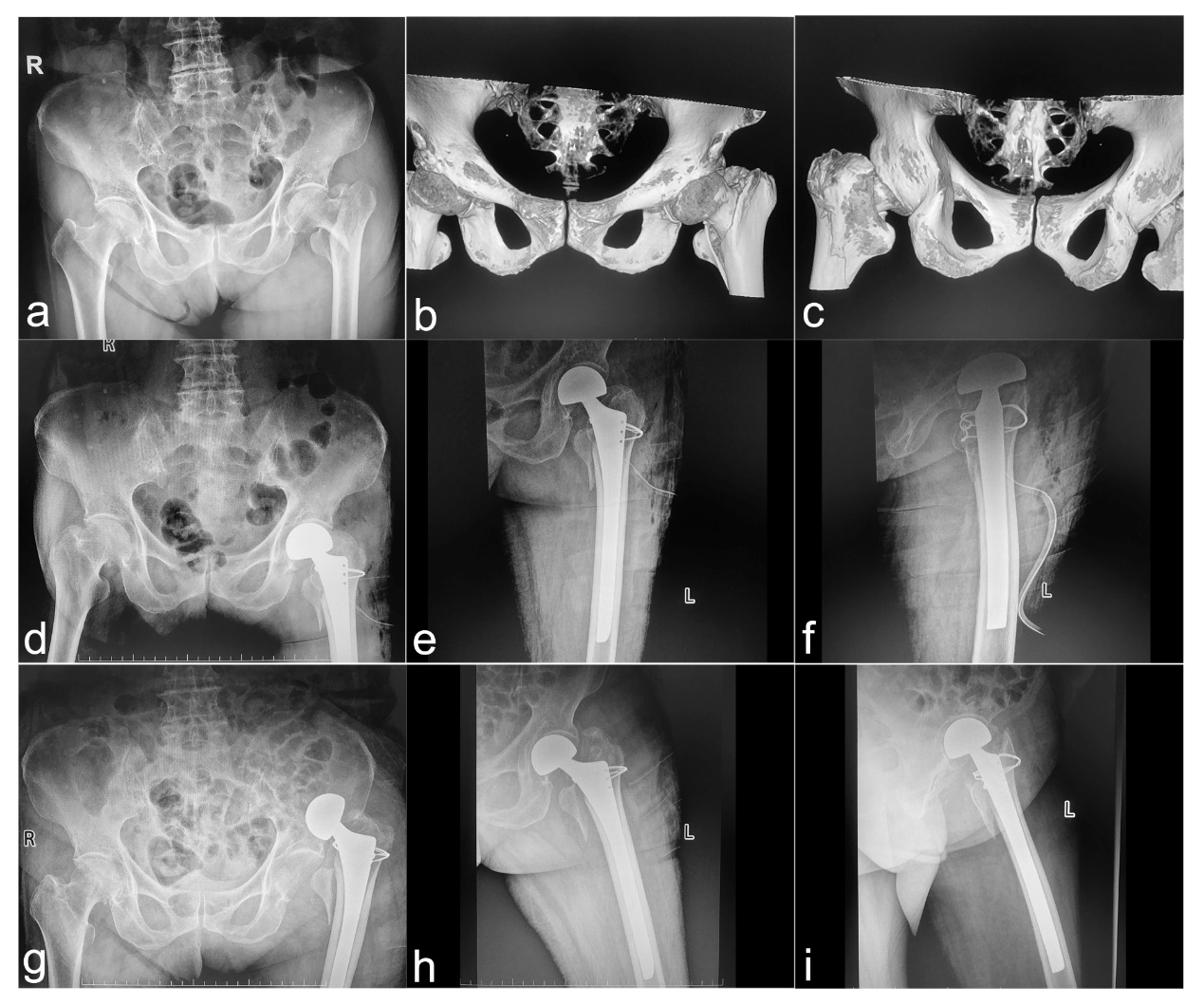


**Fig. 5.** A 81-year-old female patient with left femoral intertrochanteric fracture, caused by a fall while walking (AO classification: 31-A2.2). CBH was performed. 9 days after operation，the patient dislocated the left hip joint due to a fall. We gave her a manipulative reduction. **a.** Preoperative pelvic anteroposterior X-ray illustrating a left femoral intertrochanteric fracture. **b.** Three-dimensional CT reconstruction of the pelvis demonstrating the anteroposterior image of the left hip joint, and the lesser trochanter fracture block is clearly visible. **c.** Three-dimensional CT reconstruction of the pelvis demonstrating the posterior image of the left hip joint, and the greater trochanter fracture block is clearly visible. **d.** Postoperative pelvic anteroposterior X-ray images. **e.** Postoperative left hip anteroposterior X-ray images. **f.** Postoperative left hip lateral X-ray images. **g.** Pelvic anteroposterior X-ray showed a left hip dislocation. **h.** Pelvic anteroposterior X-ray images of the left hip joint after reduction. **i.** The lateral X-ray images of the left hip joint after reduction.
