# Supplementary figures and images for: Cementless bipolar hemiarthroplasty compared with proximal femoral nail anti-rotation of unstable intertrochanteric fractures in senile patients with osteoporosis: a retrospective study
Source: BMC Musculoskelet Disord. 2022 May 16;23:461. doi: 10.1186/s12891-022-05426-2 (PMC9112522; doi:10.1186/s12891-022-05426-2)

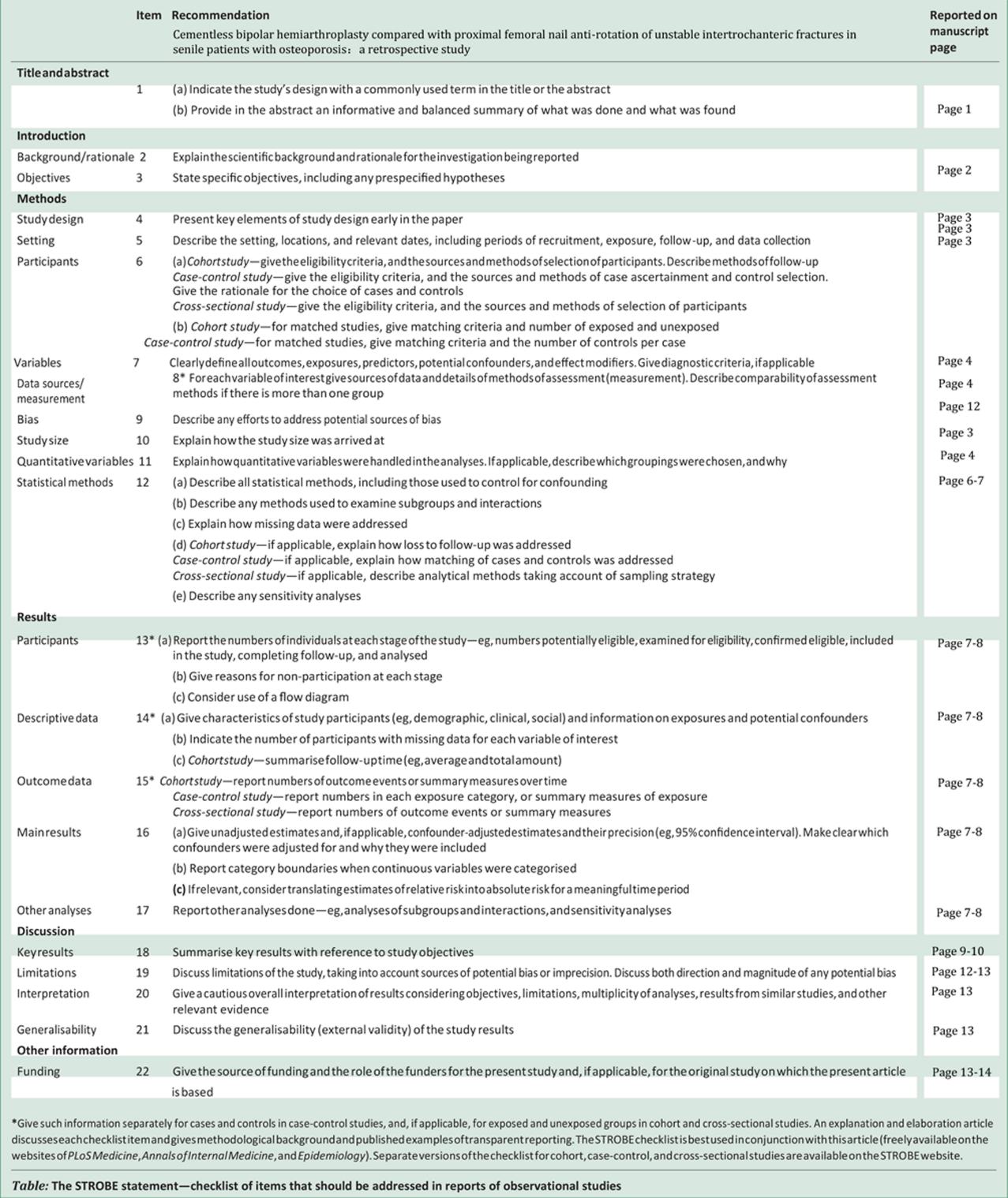


**Fig. 6.** The STROBE statement: checklist of items that should be addressed in this article.

Supplement: Supplementary file 2 — Additional file 2. [file 12891_2022_5426_MOESM2_ESM.docx]
